# Hypoxic preconditioning induces epigenetic changes and modifies swine mesenchymal stem cell angiogenesis and senescence in experimental atherosclerotic renal artery stenosis

**DOI:** 10.1186/s13287-021-02310-z

**Published:** 2021-04-14

**Authors:** Busra Isik, Roman Thaler, Busra B. Goksu, Sabena M. Conley, Hayder Al-Khafaji, Arjunmohan Mohan, Mohsen Afarideh, Abdelrhman M. Abumoawad, Xiang Y. Zhu, James D. Krier, Ishran M. Saadiq, Hui Tang, Alfonso Eirin, LaTonya J. Hickson, Andre J. van Wijnen, Stephen C. Textor, Lilach O. Lerman, Sandra M. Herrmann

**Affiliations:** 1 Division of Nephrology and Hypertension, Mayo Clinic, 200, First Street SW, Rochester, MN 55902 USA; 2 Department of Biochemistry and Molecular Biology, Rochester, USA; 3 Department of Orthopedics, Rochester, USA; 4 Division of Nephrology and Hypertension, Mayo Clinic, Jacksonville, FL USA

**Keywords:** Mesenchymal stem cells, Hypoxia, Angiogenesis, Senescence, Epigenetics, Hydroxymethylation, Chromatin organization, Adipose mesenchymal stromal cells, Atherosclerotic renal artery stenosis

## Abstract

**Background:**

Atherosclerotic renal artery stenosis (ARAS) is a risk factor for ischemic and hypertensive kidney disease (HKD) for which autologous mesenchymal stem cell (MSC) appears to be a promising therapy. However, MSCs from ARAS patients exhibit impaired function, senescence, and DNA damage, possibly due to epigenetic mechanisms. Hypoxia preconditioning (HPC) exerts beneficial effects on cellular proliferation, differentiation, and gene and protein expression. We hypothesized that HPC could influence MSC function and senescence in ARAS by epigenetic mechanisms and modulating gene expression of chromatin-modifying enzymes.

**Methods:**

Adipose-derived MSC harvested from healthy control (*N* = 8) and ARAS (*N* = 8) pigs were cultured under normoxia (20%O_2_) or hypoxia (1%O_2_) conditions. MSC function was assessed by migration, proliferation, and cytokine release in conditioned media. MSC senescence was evaluated by SA-β-gal activity. Specific pro-angiogenic and senescence genes were assessed by reverse transcription polymerase chain reaction (RT-PCR). Dot blotting was used to measure global genome 5-hydroxymethylcytosine (5hmC) levels on DNA and Western blotting of modified histone 3 (H3) proteins to quantify tri-methylated lysine-4 (H3K4me3), lysine-9 (H3K9me3), and lysine-27 (H3K27me3) residues.

**Results:**

Specific pro-angiogenic genes in ARAS assessed by RT-PCR were lower at baseline but increased under HPC, while pro-senescence genes were higher in ARAS at baseline as compared healthy MSCs. ARAS MSCs under basal conditions, displayed higher H3K4me3, H3K27me3, and 5hmC levels compared to healthy MSCs. During HPC, global 5hmC levels were decreased while no appreciable changes occurred in histone H3 tri-methylation. ARAS MSCs cultured under HPC had higher migratory and proliferative capacity as well as increased vascular endothelial growth factor and epidermal growth factor expression compared to normoxia, and SA-β-gal activity decreased in both animal groups.

**Conclusions:**

These data demonstrate that swine ARAS MSCs have decreased angiogenesis and increased senescence compared to healthy MSCs and that HPC mitigates MSC dysfunction, senescence, and DNA hydroxymethylation in ARAS MSC. Thus, HPC for MSCs may be considered for their optimization to improve autologous cell therapy in patients with nephropathies.

**Supplementary Information:**

The online version contains supplementary material available at 10.1186/s13287-021-02310-z.

## Introduction

The prevalence of chronic kidney disease (CKD) has reached epidemic proportions [1]. Hypertensive and vascular renal disease contribute to the burden of CKD. Atherosclerotic renal artery stenosis (ARAS) induces ischemic damage in the stenotic kidney and hypertensive kidney disease (HKD) in the non-stenotic kidney, leading to CKD [2]. HKD is characterized by microvascular damage, endothelial dysfunction, and loss of endogenous vasodilators, resulting in diminished renal microvasculature [3]. These injurious events lead to localized areas of hypoxia and induction of profibrotic responses, resulting in scarring and deterioration of renal function [3]. Hypertension and other vascular risk factors, including aging, smoking, susceptibility to sterile inflammation, and oxidative injury, are likely contributors to injury in small blood vessels [4]. In addition, loss of the glomerular filtration rate (GFR) impairs endogenous vasodilator functions, increases sympathetic activity, and activates the renin-angiotensin-aldosterone system (RAAS), leading to worsening hypertension [5, 6].

In HKD, the intrinsic regenerative capacity is limited. In the CKD milieu, uremic toxins may exert epigenetic and transcriptional modulation of MSC [7, 8]. Loss and dysfunction of circulating and resident reparative cells caused by oxidative stress and DNA damage may impair pro-angiogenic or anti-inflammatory functionality, promoting the development of chronic glomerulosclerosis and tubulointerstitial fibrosis [9–11]. Recent advances in regenerative cell therapies using mesenchymal stem cells (MSCs) offer the potential for renal repair for HKD patients and patients with other kidney diseases [12, 13]. MSCs are self-renewing adult somatic stem cells (i.e., non-embryonic) that can be isolated from several tissues and possess anti-fibrotic, anti-inflammatory, and pro-angiogenic paracrine activities [10, 14, 15]. We have shown that exogenous delivery of autologous adipose-tissue derived MSCs capable of increasing blood flow and GFR while reducing inflammatory biomarkers in patients with ARAS [13, 16, 17]. However, autologous patient-derived MSCs from older individuals with ARAS exhibit impaired function, senescence, and DNA damage, possibly due to epigenetic alterations [18], which might impede their reparative potential in autologous stem cell therapies.

Notably, hypoxia preconditioning (HPC) exerts beneficial effects on cellular proliferation, differentiation, and gene and protein expression [19, 20]. Aging is associated with a decrease in the regenerative potential of MSCs as well as impaired angiogenic capacities, but these effects can be counteracted by HPC [21]. We hypothesized that HPC could modify MSC function, senescence, and epigenetic patterns by modulating the gene expression levels of chromatin-modifying enzymes. In this study, we sought to evaluate whether HPC affects the function of MSCs isolated from a porcine model of ARAS. Experimentally, we examined whether HPC affects the ability of cultured MSCs to express genes linked to angiogenesis and senescence, and whether HPC can control epigenetic mechanisms associated with global patterns of DNA hydroxymethylation on the 5-position of cytidines (5-hmC) and tri-methylation (me3) of three critical histone 3 lysine (H3K) residues (i.e., H3K4me3, H3K9me3, and H3K27me3).

## Methods

### Induction of experimental swine ARAS

Domestic pigs of both sexes (initially weighing 15–17 kg) were studied during 16 weeks of observation (Fig. S1). At baseline, ARAS pigs were fed with 2% high-cholesterol diet (*N* = 8), an established surrogate for early atherosclerosis, and healthy pigs with standard chow (*N* = 8). Six weeks later, the pigs on an atherogenic diet underwent placement of a local-irritant coil in the renal artery to induce a gradual development of unilateral renal artery stenosis (RAS), as previously described [16]. Three weeks after inducing ARAS, 5–10 g of subcutaneous abdominal adipose tissue was excised in order to harvest MSCs. MSCs from matched healthy pigs were used as controls. In addition, mean arterial pressure (MAP) was directly measured through a catheter inside the carotid artery using a pressure transducer sensor. The pigs were euthanized by a terminal intravenous injection of sodium pentobarbital (100 mg/kg IV, Fatal Plus, Vortech Pharmaceuticals, Dearborn, MI, USA). All procedures in animals were approved by the Mayo Clinic Institutional Animal Care and Use Committee. Animal studies were conducted according to guidelines provided by the National Institutes of Health and the Institute of Laboratory Animal Resources, National Research Council.

### MSC culture and characterization

MSCs were isolated and expanded from abdominal adipose tissue, as previously described [16]. Following tissue harvest, the fat was immediately processed under sterile conditions by mincing and digesting in collagenase-H at 37 °C for 45 min following the tissue harvest. Serum-containing medium was added to the enzymatically digested suspension to stop the reaction. The suspension was filtered through a 100 μm cell strainer to remove the remaining tissue pieces and then centrifuged to pellet cells. Cells were resuspended in advanced minimum essential medium supplemented with 5% platelet lysate (PLTmax, Mill Creek Life Sciences, Rochester, MN, USA) and expanded in culture for 2–4 passages under each condition. MSC was characterized by imaging flow cytometry (FlowSight, Amnis, Seattle, WA, USA) to confirm expression of MSC-specific surface markers CD73 (R&D Systems®, Minneapolis, MN, USA, Cat.# AF4488), CD90 (Abcam, San Francisco, CA, USA Cat.# ab124527), and CD105 (Abcam, Cat.# ab53321). Conversely, MSC were expected to not express CD45 (Abcam, Cat. # ab51482), CD34 (BD Biosciences, San Jose, CA, Cat. # 340441), or CD14 (Abcam, Cat. # ab82012). All antibodies were used at the manufacturer’s recommended dilutions and cellular concentrations. The gating strategy for cell acquisitions was performed as previously communicated (see supplementary material) [22] Briefly, single cells were visualized by using the aspect ratio intensity and area of brightfield on the FlowSight. Determination of thresholds is assessed by observing histograms of single-stained controls (AbC™ Total Antibody Compensation Bead Kit, Molecular Probes, Eugene, OR, USA, Cat.# A10497). The appropriate gate is established, and adjustments are implemented according to the images of positively stained single cells in the scatterplot. Data was analyzed using Amnis® Image Data Exploration and Analysis Software (IDEAS version 6.2) [22, 23]. .Furthermore, we previously demonstrated the potential of porcine MSC to differentiate into adipocyte, osteocyte, and chondrocyte lineages [12, 24, 25]. MSC characterization was performed according to the International Society for Cellular Therapy (ISCT) definition [26].

### Hypoxia preconditioning protocol

Cultured MSCs were maintained (passage 2–4) under normoxic (20% O_2_, 37 °C) or hypoxic conditions (1% O_2_, 37 °C) for 48–72 h. Hypoxia was achieved by placing cells in a Modular Incubator Chamber (Billumps-Rothenberg; Del Mar, CA, USA) that was flushed with a mixture of 1% O_2_, 5% CO_2_, and 94% N_2_, confirmed by an infrared gas analyzer (Novametrics, Wallingford, CT, USA).

### MSCs in vitro functional studies

Cell viability was detected by trypan blue staining [27]. Proliferation capacity was determined using a CellTiter 96® AQ_ueous_ Non-Radioactive Cell Proliferation Assay (Promega, Madison, WI, USA, Cat. #G5421) according to the manufacturer’s instructions. MSC migration was tested using a QCM™ Colorimetric Cell Assay (EMD Millipore, Burlington, MA, USA, Cat. # ECM508), performed according to the company’s standard protocol.

### Apoptosis assay

Apoptotic cells were detected using APC Annexin V/Dead Cell Apoptosis Kit with APC Annexin V and SYTOX® Green (Invitrogen, Eugene, OR, USA cat# V35113). Cells were resuspended at a concentration of 1 × 10^6^ cells/mL in 1X Annexin-binding buffer; 5 μL APC-Annexin V and 1 μL of the 1 μM SYTOX® Green stain working solution were added to each 100 μL cell suspension. The cells were incubated at 37 °C in an atmosphere of 5% CO_2_ for 15 min, and then analyzed using FlowSight (Amnis Corp, Seattle, WA) flow cytometer; 25,000 cells from each sample were collected and subsequently analyzed using the IDEAS (Amnis Corp, Seattle, WA) Application version 6.0 [19].

### Angiogenic factors released by MSCs

Conditioned media aliquots were collected before harvesting the cells and stored at − 80 °C until assayed. Cells were counted per biologic sample following harvest and levels of angiogenic factors using porcine vascular endothelial growth factor-A (VEGF; Sigma-Aldrich, St. Louis, MO, USA, Cat.# RAB1135) and epidermal growth factor (EGF; MyBioSource, San Diego, CA, USA, Cat.# MBS703521) were measured by enzyme-linked immunosorbent assays (ELISAs) according to manufacturer protocols.

### SA-β-Gal

MSC senescence was evaluated by senescence-associated β-galactosidase (SA-β-gal) activity using a Cellular Senescence Activity Assay kit (Enzo Life Sciences, Farmingdale, NY, USA, Cat. #ENZ-KIT129-0120), performed according to manufacturer’s instruction.

### Quantitative reverse transcription PCR (RT-qPCR)

A total of 0.5–1 × 10^6^ pig MSC—were homogenized in 350ul of ice-cold lysis buffer, supplied by mirVana PARIS total RNA isolation kit (ThermoFisher Scientific, Waltham, MA, USA, Cat# AM1556). The total RNA was isolated from homogenized samples according to the kit protocol. RNA concentrations were measured by NanoDrop. First-strand cDNA was produced from 720 ng of total RNA using SuperScript VILO cDNA Synthesis kit (ThermoFisher Scientific, Cat.# 11754-050). Relative quantitative PCR was performed using TaqMan™ assays, containing 4 μl of cDNA products. All probes were purchased from Thermo Fisher scientific. Assay IDs are as follow: EGF (ss03391285), VEGF (ss03393994), P16 (APYMMFE), P21 (AJGJQY6), and GAPDH (ss03375629) was used as reference control. Negative controls with no cDNA were cycled in parallel with each run. PCR analysis was performed on an Applied Biosystems QuantStudio™ 7 instrument using the following conditions: 50 °C for 2 min, 95 °C for 10 min, and 40 cycles of 95 °C for 15 s and 60 °C for 1 min. Fold changes of gene expressions were calculated using 2-ΔΔCT method.

### Epigenetic assays

DNA extraction from MSCs under normoxic and hypoxic conditions were performed using the PureLink™ Genomic DNA Mini Kit according to the kit protocol (ThermoFisher Scientific, Cat.# K182001). The DNA concentrations were measured with an ultraviolet spectrophotometer (NanoDrop). Global DNA hydroxymethylation levels were assessed by dot blot (DB) analysis. Genomic DNA samples were prepared by diluting total DNA to final amounts of 1, 0.5, and 0.25 μg per dot in 0.1 M NaOH. The samples were denatured at 95 °C for 10 min and cooled quickly on an ice bath followed by neutralization with ammonium acetate. Samples volumes were then loaded on a nitrocellulose membrane using a Bio-Dot® Microfiltration Apparatus (Bio-Rad Laboratories) according to the manufacturer’s recommendations. The membrane was rinsed with 2X saline-sodium citrate, air-dried, and blocked with 5% skimmed milk in PBS for 1 h. The membrane was quickly washed with PBS and incubated overnight with an antibody recognizing 5-hydroxymethyl cytosine (5hmC) (Active Motif, #39791 as suggested by the supplier). The next day, the membrane was washed with PBS and incubated with an anti-rabbit IgG HRP linked secondary antibody (Cell Signaling, Danvers, MA, USA, Cat.# 7074; dilution 1:5000) in 5% skimmed milk in PBS for 1 h at room temperature. The blots were then washed with PBS and developed using the SuperSignal West Femto Maximum Sensitivity Substrate kit (Thermo Fisher Scientific) using the auto-exposure settings on the ChemiDoc™ Touch Imaging System (Bio-Rad Laboratories). Data were quantified by densitometry and analyzed using Image Lab software by applying background subtraction and approximated for linearity [28].

Cell lysis and Western blot analysis for the characterization of histone H3 tri-methylation marks were performed as described previously [28–30]. Proteins were visualized using the ECL Prime detection kit. Primary antibodies used were as follows: Anti-total H3 (1:10,000; Cat.#05-928, Millipore), anti-H3K4me3 (1:1000, Cat.#9751, Cell Signaling), anti-H3K9me3 (1:2500, Cat.#39065, Active Motif), and anti-H3K27me3 (1:5000; Cat.#17-622, Millipore), and tubulin (1:10,000; E7, University of Iowa Hybridoma Bank).

An anti-rabbit IgG HRP linked secondary antibody (Cell Signaling Cat #7074; dilution 1:5000) was used in 5% skimmed milk. The blots were developed using the SuperSignal West Femto Maximum Sensitivity Substrate kit (Thermo Fisher Scientific) using the auto-exposure settings on the ChemiDoc™ Touch Imaging System (Bio-Rad Laboratories). Data were quantified by densitometry and analyzed using Image Lab software by applying background subtraction and approximated for linearity [28].

### meDIP-seq methods

Bioinformatic analysis of meDIP-seq samples was performed by aligning paired-end sequenced FASTQs to the porcine reference genome (susScr11.1; using bowtie2 (v 2.3.3.1) [31]. Alignment files were filtered to retain properly mapped pairs in which at least one-end maps uniquely to the genome. PCR duplicates were removed using PICARD (v1.67) MarkDuplicates. The meDIP peaks were called using MACS [32] to identify punctate binding events with both high sensitivity and specificity [33].

Differential Peak Analysis was performed using the DiffBind (v2.14.0) [34] application package. This package executes differential binding analysis of ChIP-Seq, and similar epigenomics profiling techniques, peak data by comparing biological replicates from different experimental conditions and determines sites of differential 5hmC coverage. DiffBind analysis was performed on peaks that were called to be enriched by MACS, as well as the genomic coordinates for exons and promoters in Ensembl (release 98) [35]). Differential peaks and genomic coverage bins were annotated and assigned to the corresponding genes by the HOMER (v4.10) [36] peak annotation tool.

Manual analysis of 5hmC coverage was performed using per-base coverage of regions of interest, calculated by bedtools (v2.20.0) genomeCoverageBed. Sequence read values for the overall exonic 5hmC coverage per gene were also calculated in parallel using htseq-count (v0.9.1) [37]. These faux-RNA counts 5hmC coverage were then processed by edgeR (v3.28.1) [38] to obtain differences in read frequencies for genomic 5hmC coverage at exons analogous to differential expression analysis of RNA reads.

### Angiogenic and senescence gene analysis in MSCs

To elucidate whether ARAS regulates gene 5 hmC levels and to monitor the effects of hypoxia, we used the MGI database (http://www.informatics.jax.org/genes.shtml) to screen genes associated with angiogenic and senescence signaling. Levels of 5hmC < 0.7 and fold changes in the ratio of hypoxia and normoxia > 1.4 were considered of biological interest and *p* values < 0.05 were considered significantly different. Heat maps were created using Morpheus (https://software.broadinstitute.org/morpheus/).

### Statistical analysis

Data are represented as mean ± standard deviation and analyzed using JMP 14.1 software. The normality assumption was tested using the Shapiro-Wilk Test. Two-sample or Wilcoxon tests were used for comparisons between groups as appropriated. Differences between the epigenetic marks of healthy and ARAS MSCs, as well as clinical characteristics of experimental pigs, were tested using the two-sample or Wilcoxon tests. *p* value of < 0.05 was considered statistically significant.

## Results

Baseline characteristics of healthy and ARAS pigs used in this study are summarized in Table 1. Weight and heart rate were not different between groups, but diastolic blood pressure (DBP), mean arterial pressure (MAP), serum creatinine, and cholesterol were higher in ARAS pigs.

**Table 1:** Characteristics of healthy and ARAS pigs

Study	Healthy pigs(***N*** = 8)	ARAS pigs(N = 8)	*P* value
Weight (kg)	60.8 ± 6.8	62.8 ± 6.5	0.6
Heart rate (beats per minute)	87.1 ± 24.2	89.4 ± 18.4	0.8
Systolic blood pressure (mmHg)	120 ± 12.4	134 ± 16	0.08
Diastolic blood pressure (mmHg)	81.3 ± 13.1	100.4 ± 12.8	0.01
Mean arterial pressure (mmHg)	94.3 ± 12.1	111.6 ± 13.7	0.02
Serum creatinine (mg/dL)	1.4 ± 0.2	2.5 ± 0.4	0.0009
Cholesterol (mg/dL)	77.1 ± 5.5	588 ± 104.3	< 0.0001

### Functional effects of ARAS on swine MSCs under normoxic and hypoxic conditions

**Fig. 1:**
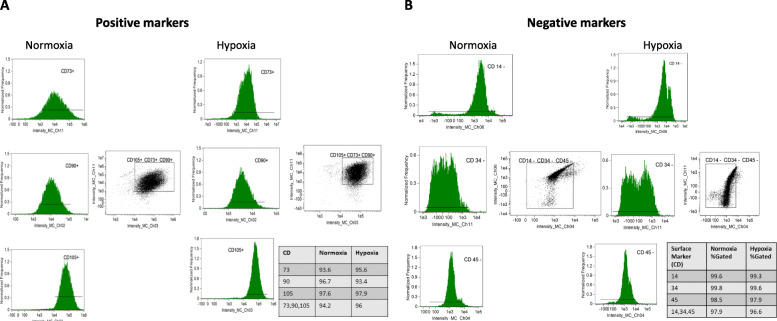
Mesenchymal stem cell (MSC) characterization in normoxic and hypoxic conditions. **a** Positive MSC markers (CD105, CD73, and CD90) and **b** negative MSC markers (CD,14, CD34, CD45)

**Fig. 2:**
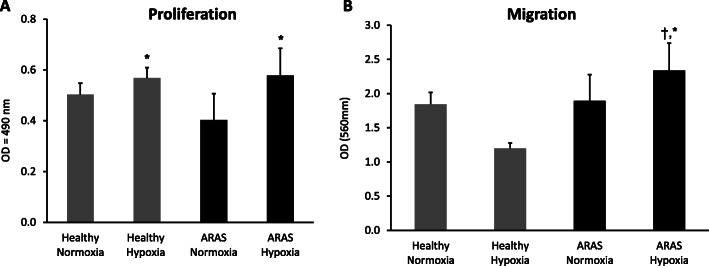
Functional differences between healthy and ARAS MSCs under normoxic and hypoxic conditions. Proliferation was increased under hypoxic conditions in both healthy and ARAS MSC (**a**) while migration was only improved in ARAS MSC under hypoxic preconditioning (**b**). *p < 0.05 hypoxia vs normoxia. ^†^*p* < 0.05 ARAS vs healthy

MSCs harvested from adipose tissue of both healthy and ARAS pigs retained characteristic plastic-adherent, fibroblast-like morphology, expressed CD90, CD73, and CD105 markers, but not CD45, CD14, or CD34 markers, under either normoxia and hypoxia (Fig. 1a, b). The cellular morphology was similar in both groups, and their multi-lineage potential was further confirmed by differentiation into adipocytes, chondrocytes, and osteocytes (Fig. S2A, B). MSC migration and proliferation capacity were not different between ARAS-MSCs and healthy MSCs at baseline normoxic conditions. However, following HPC exposure, both migration (*p* = 0.017) and proliferation (*p* = 0.015) were increased in ARAS MSCs, while in healthy MSCs only proliferation was increased (*p* = 0.047) (Fig. 2a, b and Fig. S3). Furthermore, there were no differences in percentage of viable or apoptotic cells (measured by Annexin V) between the groups at baseline normoxic conditions or any change with HPC (Fig. S4).

### Hypoxia upregulated pro-angiogenic genes and posttranscriptional protein expression in MSCs

To investigate the levels of paracrine angiogenic factors secreted by MSCs, we measured VEGF and EGF expression in MSCs cultured under normoxic and hypoxic conditions.

**Fig. 3:**
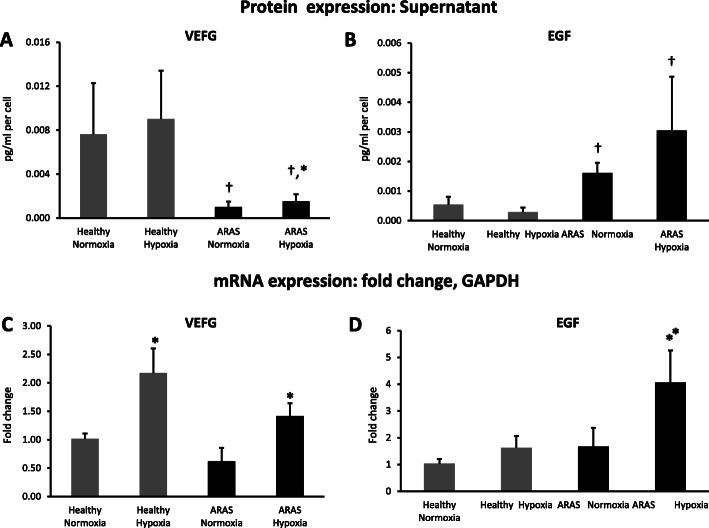
Hypoxia upregulated pro-angiogenic protein and gene expression in MSC. VEGF release by swine MSC was low in ARAS compared to healthy pigs and augmented but not normalized during hypoxia (**a**). EGF secretion was elevated in ARAS in comparison to healthy MSC (**b**). Hypoxia upregulated pro-angiogenic genes (VEGF-a) in healthy and ARAS MSC (**c**), as well as EGF gene expression in ARAS (D). *p < 0.05 hypoxia vs normoxia. ^†^*p* < 0.05 ARAS vs healthy

VEGF release by ARAS MSCs under baseline normoxic conditions was markedly lower compared to healthy controls (*p* = 0.002), whereas EGF levels were higher both at baseline (*p* = 0.01) and during hypoxia (*p* = 0.004) compared to healthy MSC (Fig. 3a, b). When ARAS MSCs were exposed to hypoxia, VEGF release increased (*p* = 0.015), but not to the level observed in healthy porcine MSCs. Overall, there was no change in angiogenic factor release by healthy MSCs exposed to hypoxia.

At the gene level, we found that VEGF and EGF mRNA expression was not different between healthy and ARAS MSCs at baseline normoxic conditions. Under hypoxia, VEGF gene expression was increased in both healthy (*p* = 0.03) and ARAS (*p* = 0.035) groups, whereas EGF expression only increased in ARAS-MSCs (*p* = 0.02) (Fig. 3c, d).

### Global DNA hydroxymethylation (5hmC) and histone lysine tri-methylation (H3Kme3) marks in healthy and ARAS swine MSCs under basal conditions

**Fig. 4:**
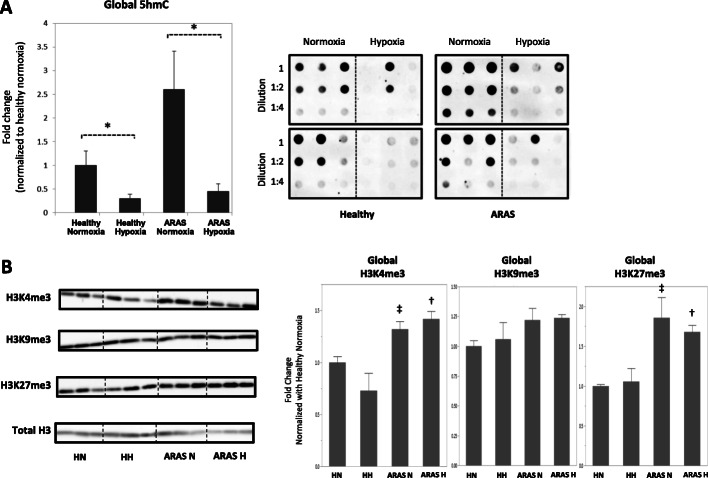
Global epigenetic alterations in ARAS (*N* = 4) compared to healthy MSCs (*N* = 5) under normoxic and hypoxic conditions demonstrated by: Dot-blotting showing MSC DNA hydroxymethylation measuring genomic 5-hydroxymethyl-cytosine (5hmC). **a** Western blotting of histone-3 protein measuring genomic trimethylation on lysine-4 (H3K4me3), 9 (H3K9me3), and 27 (H3K27me3) residues. **b** Genomic 5hmC was reduced during HPC, while H3K4me3 and H3K27me3 marks in ARAS were elevated as compared to healthy controls at baseline and during HPC. No differences in H3K9 levels. HN healthy normoxia, HH healthy hypoxia, ARAS-N ARAS normoxia, ARAS-H ARAS hypoxia. *p < 0.05 ARAS hypoxia vs ARAS normoxia. #*p* < 0.05 healthy hypoxia vs healthy normoxia. ^‡^*p* < 0.05 ARAS normoxia vs healthy normoxia. ^†^*p* < 0.05 ARAS hypoxia vs healthy hypoxia

We examined global 5hmC levels and trimethylation of H3 lysine 4 (H3K4me3) because these epigenetic marks are generally associated with gene activation, while trimethylation of H3 lysine 27 (H3K27me3) and H3 lysine 9 (H3K9me3) was analyzed because these marks are linked to gene suppression. Each of these epigenetic marks is dynamic and responds to both intra- and extracellular cues. The levels of 5hmC in genomic DNA tended to be higher in ARAS MSCs but did not reach statistical significance when compared to healthy MSCs (*p* = 0.14) under basal normoxic conditions (Fig. 4a). This finding is generally consistent with alterations in epigenetic marks due to disease conditions, but the dynamic nature of these epigenetic changes may increase intra-group variation and compromise probability values. Furthermore, we observed increased global H3K4me3 and H3K27me3 marks in ARAS MSCs relative to healthy swine MSCs (*p* = 0.03 and *p* = 0.006, respectively) at baseline (Fig. 4b). By contrast, the average global levels of H3K9me3 were comparable between healthy and ARAS swine MSC samples. Hence, ARAS MSCs appear to exhibit selective differences in global epigenetic marks under basal conditions.

### Effects of HPC on epigenetic DNA hydroxylation and histone methylation marks in swine healthy and ARAS MSCs

HPC led to a significant reduction in global 5hmC in both ARAS and healthy swine MSCs with overall reductions of 82% and 70%, respectively (Fig. 4a) (*p* = 0.04 in both groups), but had no effects on H3K4me3, H3K9me3, and H3K27me3 marks in either group (Fig. 4b). Hence, hypoxia may selectively reduce hydroxymethylation levels near genes that are inhibited by DNA methylation, which is predicted to reinforce the suppressed epigenetic state of the genes in the corresponding genomic regions.

### Effects of HPC on senescence in swine healthy and ARAS MSCs

**Fig. 5:**
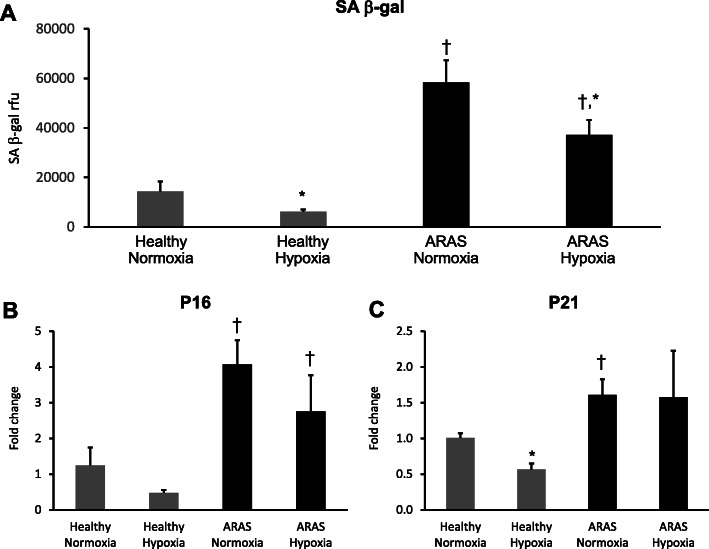
Mesenchymal stem cell senescence decreases under hypoxic preconditioning. Saβ-gal activity levels were increased in ARAS as compared to healthy MSCs and it decreased its activity in both groups during HPC (**a**). Similarly senescent genes P16 and P21 mRNA expression was increased in ARAS as compared to healthy MSCs at baseline (**b**, **c**, respectively), but HPC only decreased P21 in healthy MSCs (**c**). HPC hypoxia pre-conditioning. *p < 0.05 hypoxia vs normoxia. ^†^*p* < 0.05 ARAS vs healthy

Because atherosclerosis and the CKD milieu in HKD may cause senescence and alter functionality of MSC, we evaluated if cellular senescence was influenced by HPC. At baseline normoxic conditions, senescence-associated β-galactosidase (SA-β-gal) activity was almost fourfold higher in ARAS compared to healthy MSCs (*p* = 0.001). Once MSCs were exposed to hypoxia this difference persisted, but SA-β-gal activity decreased significantly in both healthy (*p* = 0.05) and the ARAS (*p* = 0.005) groups (Fig. 5a). These baseline differences were also seen when analyzing mRNA expression of P16 (*p* = 0.006) and P21 *(p* = 0.02), which were significantly higher in ARAS as compared to healthy MScs. Furthermore, HPC decreased P21 mRNA expression in healthy MSCs (*p* = 0.005) (Fig. 5b, c).

### Hypoxia affects DNA hydroxymethylation of angiogenic and senescence-related genes differently in healthy compared to ARAS MSCs

**Fig. 6:**
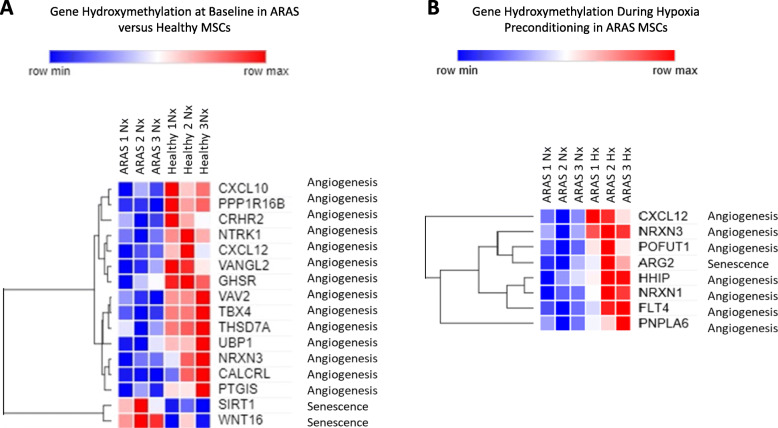
Comparison of meDIPseq genomic distribution of 5hmC heat maps of ARAS (*N* = 3) and healthy (*N* = 3) MSCs. Heat maps of 5hmC average are peaks upregulated in healthy MSC as compared vs ARAS MSCs at baseline (**a**) and effect of HPC in angiogenic and senescence genes (**b**) in ARAS MSCs. Heat maps of 5hmC average are peaks upregulated in healthy MSCs as compared vs ARAS MSCs (**a**) and effect of HPC in angiogenic and senescence genes (**b**) in ARAS MSCs. HPC hypoxia pre-conditioning

The genomic distribution of 5hmC was examined with emphasis on angiogenic and senescence genes having significant average peaks. At baseline under normoxic conditions, angiogenic regions were differentially hydroxymethylated in healthy versus ARAS MSCs. Compared to ARAS MSCs, healthy MSCs exhibited higher average peaks (*p* < 0.05) predominantly in the intergenic regions of angiogenic genes, such as CXCL10, VAV2, TRK1, TBX4,VANGL2, THSD7A, PPP1R16B, NRXN, CRHR2, CXCL12, UBP1, PTGIS, CALCRL, and GHSR (Fig. 6a). In addition, the senescence related genes SIRT1 and WNT16 displayed significantly elevated average 5hmC peaks in ARAS compared to healthy MSCs (*p* < 0.05) (Fig. 6a). Interestingly, hypoxic conditioning of ARAS MSC reduced the number of angiogenic genes with elevated levels of hydroxymethylation, with only seven genes showing enhanced 5hmC levels (*p* < 0.05; HHIP, CXCL12, NRXN3, POFUT1, PNPLA6, NRXN1, FLT4). Furthermore, examination of the levels and distribution of 5hmC across the entire genome, ARG 2 was the only senescence gene that displayed significant changes reflected by elevation of the average 5hmC peaks in ARAS compared to healthy MSC during hypoxia (Fig. 6b).

## Discussion

The key findings of this study are decreased angiogenic capacity, as evident in decreased levels of VEGF, and increased cellular senescence, as reflected by increased senescence-associated SA-β-gal activity, P16 and P21, in adipose tissue-derived MSCs obtained from ARAS pigs compared to controls. Importantly, these unfavorable alterations were substantially reversed by HPC without affecting apoptosis. Furthermore, this study showed for the first time unique epigenetic traits of ARAS MSCs, which compared to healthy MSCs displayed higher levels of global 5hmC (reflecting active open chromatin), as well as gene activating marks H3K4me3 marks and gene suppressive H3K27me3 marks, but comparable levels of gene suppressive marks such as H3K9me3. These changes reflect global modifications in the manner by which DNA is spatially organized in the nucleus to support or suppress gene expression. Interestingly, HPC improved MSC proliferation, migration, and angiogenic capacity and reduced senescence burden, consistent with epigenetic changes in DNA hydroxymethylation and histone trimethylation in selected sets of genes. While our data suggest that ARAS MSCs have a clearly distinct epigenetic state that is altered by HPC, in the current analysis, the dynamic and intricate nature of epigenetic modifications does not yet permit direct correlation of the activation or suppression of specific genes relevant to regenerative functions of MSCs.

MSCs obtained from patients with ARAS exhibit impaired function with decreased expression of angiogenic proteins, reduced migration capacity, and increased senescence and senescence-associated DNA damage compared to MSC from healthy individuals [19]. This dysfunction might be observed in aging but also magnified by disease processes such as CKD and cardiovascular risk factors. Their MSCs exhibited greater levels of senescence-associated DNA damage and reduced migration capacity as compared to MSC from healthy individuals, associated with decreased expression of angiogenic proteins [19]. Therefore, a pro-atherosclerotic milieu and chronic diseases such as HKD very likely also restrict the effectiveness of autologous MSCs as a therapeutic approach in these patients. The current study recapitulates these findings and shows that major MSC dysregulation can develop in hypertensive swine with dyslipidemia and moderate kidney dysfunction without the effects of aging per se. Furthermore, our study extends observations that MSCs from a CKD mouse model exhibit decreased expression of VEGF, as well as increased cellular senescence and decreased proliferation capacity [39]. Accordingly, CKD and cardiovascular risk factors may decrease the functional capacities (and therapeutic utility) of MSC by acting as cysto-stressors via oxidative stress, circulating cytokines, and genetic and epigenetic alterations [18, 19, 40]. The latter has been implicated in a range of processes including atherosclerosis, hypertension, CKD, obesity, and aging [18, 40–44].

Epigenetic changes associated with ARAS and hypoxic condition include alterations in 5hmC [45, 46] which represents a stable epigenetic mark that promotes gene transcription during development and cellular differentiation (rather than just an intermediate product during DNA demethylation process) [47]. In our study in a pre-clinical model of ARAS, we found that MSCs have elevated basal global 5hmC levels compared to healthy MSCs. Furthermore, we identified 14 angiogenic genes with decreased average 5hmC peak levels in MSCs from ARAS pigs, whereas for the senescence-associated genes SIRT1 and WNT16, hydroxymethylation was increased compared to healthy MSCs. This finding correlates with enrichment of 5hmC on the gene bodies, which has been associated with activation of transcription [48].

Global increase in hydroxymethylation in ARAS MSCs was accompanied by different global histone H3 methylation levels in these groups of cells. ARAS MSCs displayed increased H3K4me3 and H3K27me3 marks, whereas levels of H3K9me3 were comparable to those in healthy MSCs. In general, transcription start sites of actively transcribed genes are marked by H3K4me3 while gene repression can be mediated through two distinct mechanisms involving H3K9me3 and H3K27me3 [49]. In ARAS MSCs, the shift in both active (5hmC and H3K4me3) and repressive (H3K27me3) epigenetic marks may reflect a divergence in normal gene expression and thus partially account for the altered cellular proprieties found in these cells. The observed cell type–specific differences in epigenomic profiles between ARAS and healthy MSCs suggests that epigenomic profiles have potential value as novel molecular diagnostics to understand what types of exposures and disease conditions may render MSCs suitable or unsuitable for therapeutic use.

Oxygen is essential for mammalian metabolism and physiological functions because of its use in cellular energy production and cofactor/substrate for many enzymes including DNA 5mC hydroxylases. When exposed to HPC, widespread global epigenetic changes occurred in both ARAS and healthy MSCs, with a decrease in global hydroxymethylation, but no changes were observed in global histone tri-methylation. In ARAS MSCs, levels of 5hmC were enhanced in 6 genes, with only one senescent gene (ARG2) showing increased levels of 5hmC. Hypoxia provokes a dramatic reduction in 5hmC levels, and changes in the histone methylation status at promoters of hypoxia-inducible genes [50]. One key finding of this study is that hypoxic conditioning selectively alters at least one (i.e., 5-hmC) of the many possible epigenetic DNA and histone modifications which are interpreted by transcriptional factors and co-factors to balance transcription [51].

MSCs mode of action involves a strong paracrine component resulting from active factors secreted in response to the local microenvironment [15, 52]. Previous studies have applied HPC to MSCs as a strategy to enhance stem cell survival in vitro, proliferation, and repair capacity [53–55]. Indeed, restoring MSC paracrine effects through HPC may improve immunomodulatory, angiogenic, antiapoptotic effects, and promote activation of local quiescent stem cells [56]. In our study, HPC improved MSC function, reflected in increased proliferation in healthy and ARAS MSCs, upregulated angiogenic factors, and downregulated cellular senescence. The results of this study identify considerable functional deficiencies in MSCs from ARAS compared to healthy MSCs. Therefore, preconditioning, genetic modification, and optimization of MSC culture conditions can be useful to improve MSC functions in vitro and support their clinical application in regenerative medicine.

This study is limited by the small sample size of ARAS and healthy pigs. Second, 5hmC originates from oxidation of 5-methylcytosine (5-mC) by the TET family of proteins. Decreased TET activity reduces hydroxylation of 5mC to 5hmC, and formation of 5hmC levels is determined by oxygen availability [57]. Changes at the level of individual genes are in part a reflection of global levels of hydroxymethylation and histone trimethylation, but our observations do not directly explain changes induced by hypoxia on angiogenic protein expression or senescence activity. However, there are many transcriptional and post-transcriptional mechanisms (e.g., microRNA levels) that work in parallel with the observed epigenetic changes at the level of chromatin that could obscure straightforward cause and effect relationships. Future studies are required to characterize in detail how HPC improve ARAS dysfunction beyond the epigenetic findings.

In conclusion, we showed a selective increase in H3K4me3 and H3K27me3 marks and unchanged H3K9me3 marks in ARAS MSCs compared to healthy MSCs. These dynamic global epigenetic changes suggest that cells under ARAS conditions are actively balancing the transcriptional repression and activation of different sets of genes that are linked to the regenerative functions of MSCs. The epigenetic differences may relate to dysfunction of MSCs when isolated from patients with renal artery stenosis and related renal disease and limit their efficacy for autologous cell therapy. HPC has a favorable impact on global DNA hydroxymethylation and histone tri-methylation on MSCs in the setting of an experimental model of atherosclerotic renal artery stenosis. Our study therefore offers an optimistic outlook in which HPC may mediate broad epigenetic changes in DNA hydroxymethylation of MSCs that could potentially revert the epigenetic state of ARAS to a state that favors biological outcomes of autologous cell therapy.

## Supplementary Information

**Additional file 1.** Bioinformatic raw data ARAS vs Healthy MSCs during hypoxia and normoxia conditions.

**Additional file 2.** Bioinformatic raw data ARAS MSCs during hypoxia and normoxia conditions.

**Additional file 3: Supplemental Figure 1.** Schematic of the experimental protocol. RAS: renal artery stenosis, ARAS: atherosclerotic RAS, MSC: mesenchymal stem cells. **Supplemental Figure 2.** MSC Morphology and differentiation. (A) Adipose tissue-derived MSC harvested from healthy and ARAS pigs show typical morphological appearance under the microscope as spindle-shaped, fibroblast-like cells in culture.(B) Tri-lineage differentiation into adipocyte (FABP4), chondrocyte (Aggrecan), and osteocyte (Osteocalcin) lineages is achieved in both MSC groups (scale bar 200 μm). MSC: mesenchymal stem cells, ARAS: atherosclerotic renal artery stenosis, DAPI: nuclear stain, FABP4: positive staining represents adipocytes, chondrocytes, Osteocalcin: positive staining represents osteocytes, Aggrecan: positive staining represents. **Supplemental Figure 3.** Cell Migration. Cells were seeded in MSC growth medium (Advanced MEM) at 300,000 cells/cm^2^ in the top chamber of The MILLIPORE QCMTM 24-well Cell Migration Assay (ECM508) kit (Sigma-Aldrich, Billerica, MA, USA). After overnight culture either in normoxic or hypoxic conditions, the migrated cells were fixed with 1% paraformaldehyde and stained with the cell stain provided with the kit. Nonmigrated cells were removed from the upper side of the membrane. Migrated cells on the bottom were directly visualized under the microscope. **Supplemental Figure 4.** Apoptosis Assay. Representative flow cytometry scatterplots of MSC viability during normoxic and hypoxic conditions. MSC viability tested using Annexin V and Sytox shows MSC viability from healthy vs. ARAS pigs. This is a representative image where red panel represents live cells, orange panel represents dead cells, and green panel represents apoptotic cells. (A) The percentage of live and apoptotic cells were similar in both groups in either condition. MSC: mesenchymal stem cells, ARAS: atherosclerotic renal artery stenosis.

## Data Availability

The data generated or analyzed during this study are included in this published article.

## Abbreviations

ARAS: Atherosclerotic renal artery stenosis
HKD: Hypertensive kidney disease
HPC: Hypoxia preconditioning
MSC: Mesenchymal stem/stromal cells
EGF: Epidermal growth factor
VEGF: Vascular endothelial growth factor
GFR: Glomerular filtration rate
TET: Ten-eleven translocation family protein
