# Supplementary material for: Hypoxic preconditioning induces epigenetic changes and modifies swine mesenchymal stem cell angiogenesis and senescence in experimental atherosclerotic renal artery stenosis
Source: Stem Cell Res Ther. 2021 Apr 14;12:240. doi: 10.1186/s13287-021-02310-z (PMC8048283; doi:10.1186/s13287-021-02310-z)
Supplement: Supplementary file 3 — Additional file 3: Supplemental Figure 1. Schematic of the experimental protocol. RAS: renal artery stenosis, ARAS: atherosclerotic RAS, MSC: mesenchymal stem cells. Supplemental Figure 2. MSC Morphology and differentiation. (A) Adipose tissue-derived MSC harvested from healthy and ARAS pigs show typical morphological appearance under the microscope as spindle-shaped, fibroblast-like cells in culture.(B) Tri-lineage differentiation into adipocyte (FABP4), chondrocyte (Aggrecan), and osteocyte (Osteocalcin) lineages is achieved in both MSC groups (scale bar 200 μm). MSC: mesenchymal stem cells, ARAS: atherosclerotic renal artery stenosis, DAPI: nuclear stain, FABP4: positive staining represents adipocytes, chondrocytes, Osteocalcin: positive staining represents osteocytes, Aggrecan: positive staining represents. Supplemental Figure 3. Cell Migration. Cells were seeded in MSC growth medium (Advanced MEM) at 300,000 cells/cm2 in the top chamber of The MILLIPORE QCMTM 24-well Cell Migration Assay (ECM508) kit (Sigma-Aldrich, Billerica, MA, USA). After overnight culture either in normoxic or hypoxic conditions, the migrated cells were fixed with 1% paraformaldehyde and stained with the cell stain provided with the kit. Nonmigrated cells were removed from the upper side of the membrane. Migrated cells on the bottom were directly visualized under the microscope. Supplemental Figure 4. Apoptosis Assay. Representative flow cytometry scatterplots of MSC viability during normoxic and hypoxic conditions. MSC viability tested using Annexin V and Sytox shows MSC viability from healthy vs. ARAS pigs. This is a representative image where red panel represents live cells, orange panel represents dead cells, and green panel represents apoptotic cells. (A) The percentage of live and apoptotic cells were similar in both groups in either condition. MSC: mesenchymal stem cells, ARAS: atherosclerotic renal artery stenosis. [file 13287_2021_2310_MOESM3_ESM.pdf]

Table of Contents

Supplemental method: ***Gating strategy for flow Cytometry***

Using the aspect ratio intensity and area of the brightfield channel on the Amnis Flowsight we were able to visualize and gate the single cells. From here we use thresholds on the Amnis flowsight that are determined by looking at the histograms generated by the single-color stained positive and negative beads. Using this information, we were able to determine baseline thresholds to use when running our cell samples. Using the thresholds generated by the control beads, we are able to make refined adjustments based off of what we are able to visualize in the images of the cells in the scatterplot, this allows us to carefully determine the threshold which the single cells are positively stained.

Supplemental Figure 1: Schematic of the experimental protocol.

RAS: renal artery stenosis, ARAS: atherosclerotic RAS, MSC: mesenchymal stem cells

Supplemental Figure 2: MSC Morphology and differentiation.

(A) Adipose tissue-derived MSC harvested from healthy and ARAS pigs show typical morphological appearance under the microscope as spindle-shaped, fibroblast-like cells in culture.(B) Tri-lineage differentiation into adipocyte (FABP4), chondrocyte (Aggrecan), and osteocyte (Osteocalcin) lineages is achieved in both MSC groups (scale bar 200 μm). MSC: mesenchymal stem cells, ARAS: atherosclerotic renal artery stenosis, DAPI: nuclear stain, FABP4: positive staining represents adipocytes, chondrocytes, Osteocalcin: positive staining represents osteocytes, Aggrecan: positive staining represents

Supplemental Figure 3: Cell Migration

Cells were seeded in MSC growth medium (Advanced MEM) at 300,000 cells/cm^2 in the top chamber of The MILLIPORE QCMTM 24-well Cell Migration Assay (ECM508) kit (Sigma-Aldrich, Billerica, MA, USA).After overnight culture either in normoxic or hypoxic conditions, the migrated cells were fixed with 1% paraformaldehyde and stained with the cell stain provided with the kit. Nonmigrated cells were removed from the upper side of the membrane. Migrated cells on the bottom were directly visualized under the microscope.

Supplemental Figure 4: Apoptosis Assay

Representative flow cytometry scatterplots of MSC viability during normoxic and hypoxic conditions. MSC viability tested using Annexin V and Sytox shows MSC viability from healthy vs. ARAS pigs. This is a representative image where red panel represents live cells, orange panel represents dead cells, and green panel represents apoptotic cells. (A) The percentage of live and apoptotic cells were similar in both groups in either condition. MSC: mesenchymal stem cells, ARAS: atherosclerotic renal artery stenosis

Figure S1

Animal ARAS Study Protocol

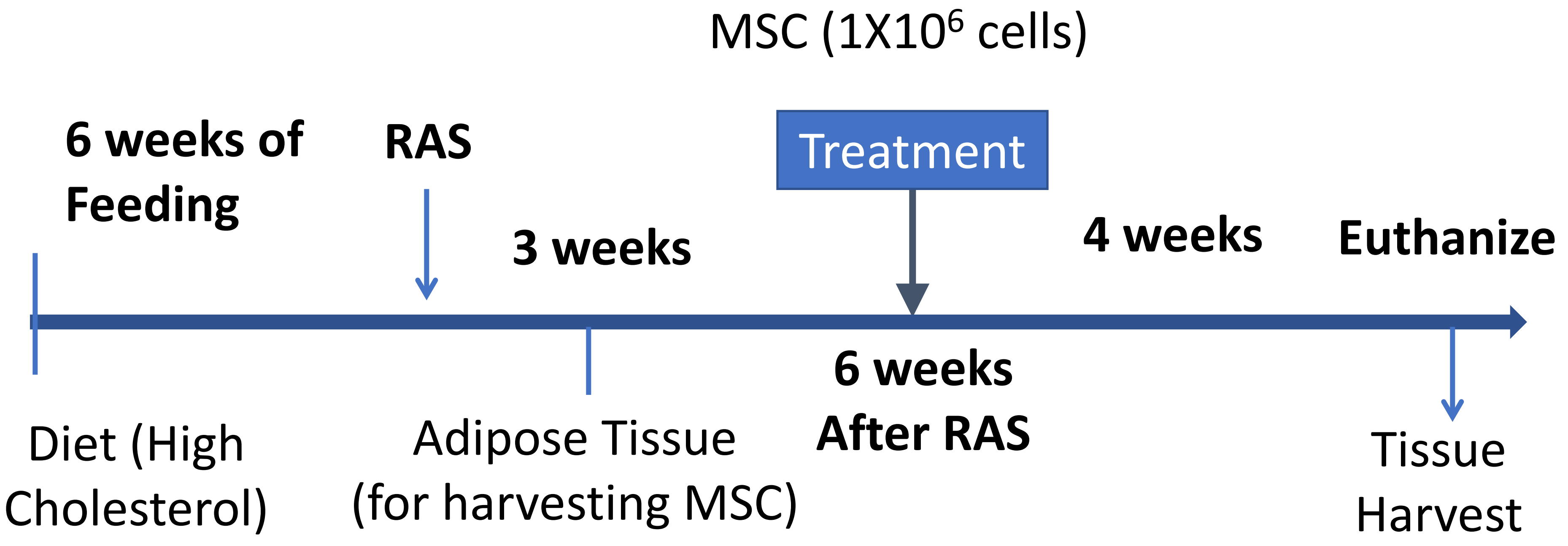

Figure S2

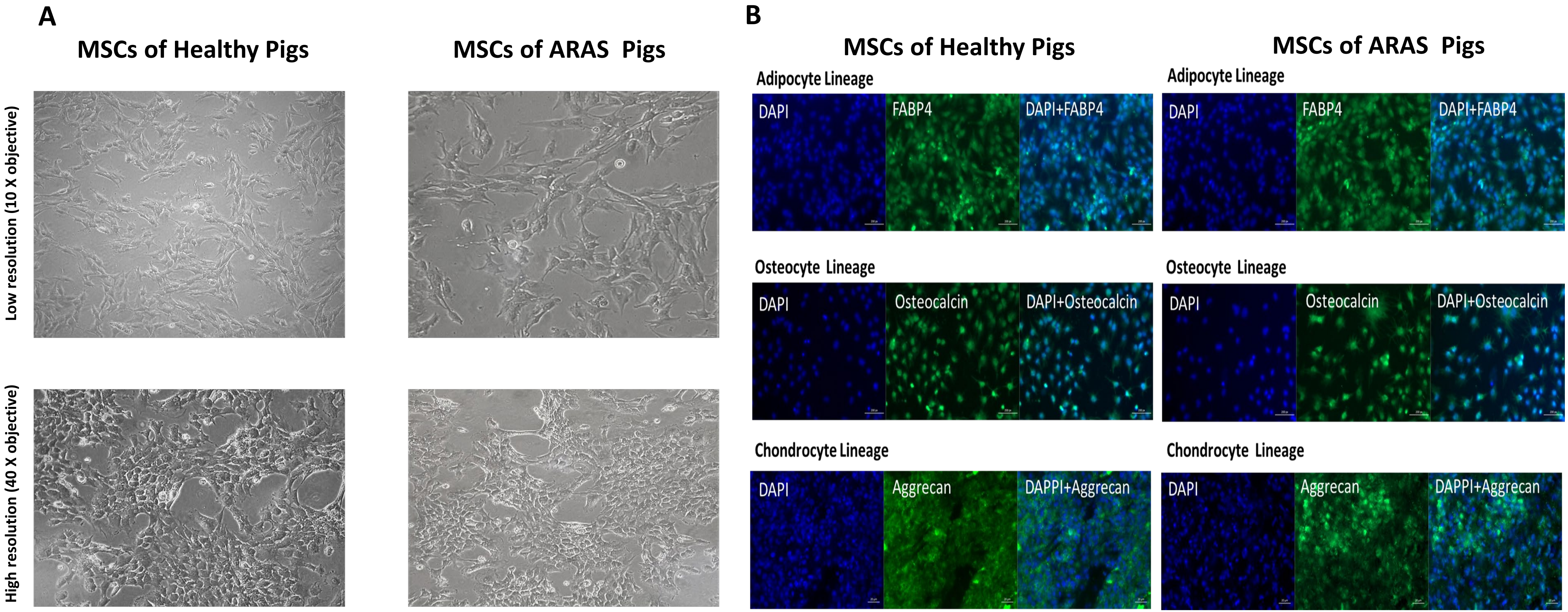
A
MSCs of Healthy Pigs
MSCs of ARAS Pigs
Low resolution (10 X objective)
High resolution (40 X objective)
B
MSCs of Healthy Pigs
MSCs of ARAS Pigs
Adipocyte Lineage
DAPI
FABP4
DAPI+FABP4
Osteocyte Lineage
DAPI
Osteocalcin
DAPI+Osteocalcin
Chondrocyte Lineage
DAPI
Aggrecan
DAPPI+Aggrecan

Figure S3

Normoxia — Low resolution (5 X objective)

Hypoxia — Low resolution (5 X objective)

A MSCs of Healthy Pigs

B MSCs of ARAS Pigs

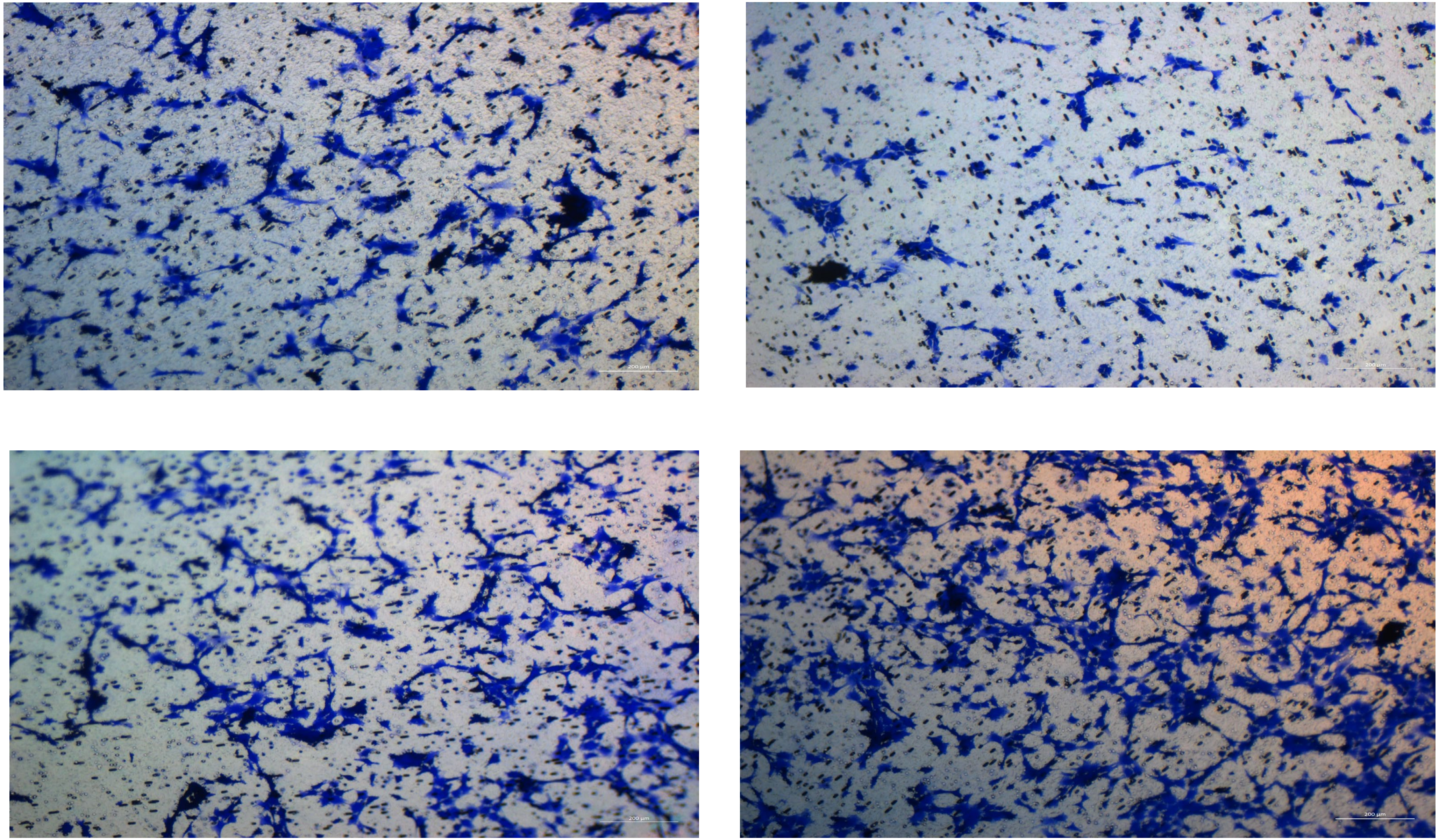

Figure S4

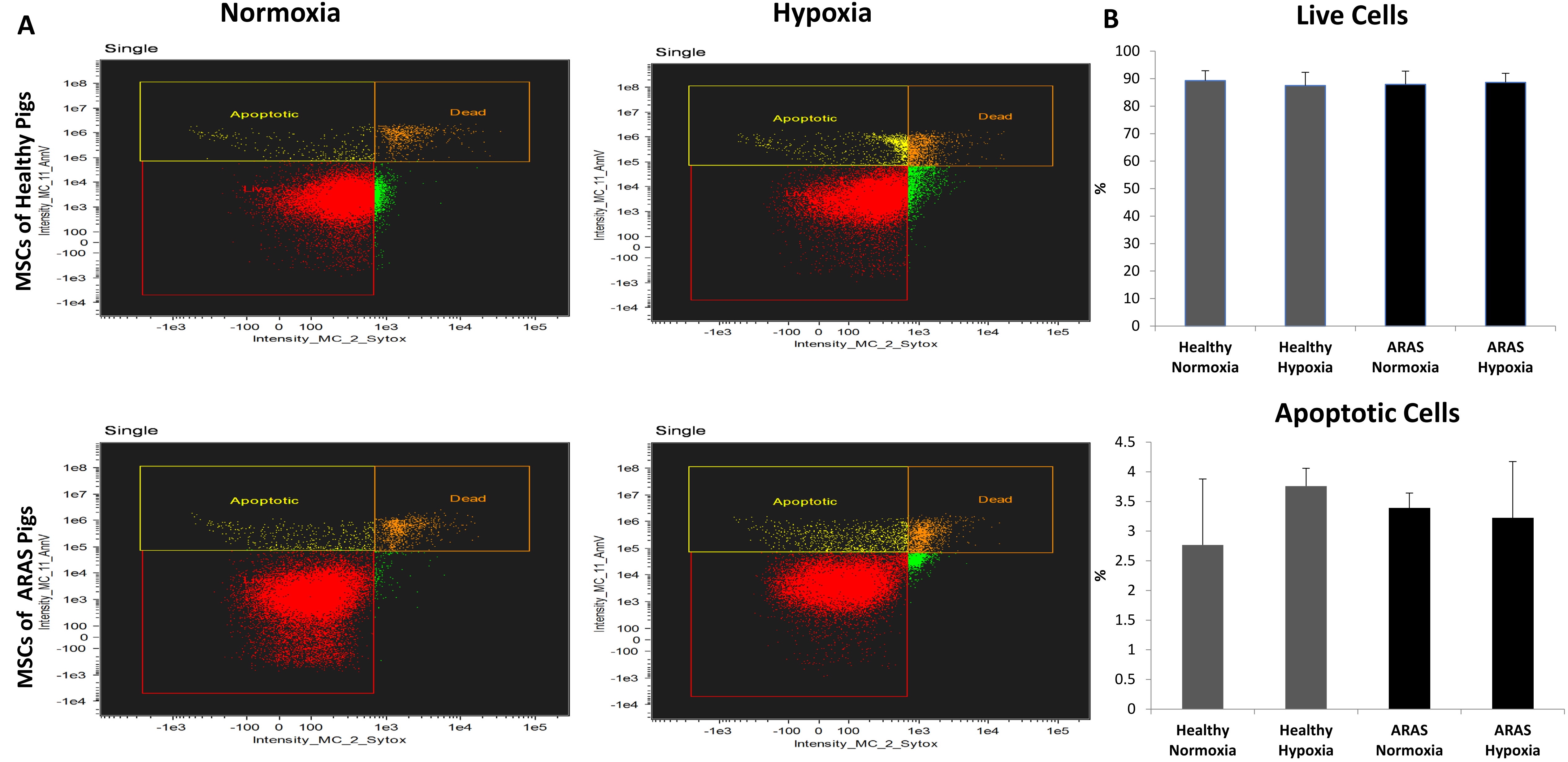

A
Normoxia
Hypoxia
MSCs of Healthy Pigs
MSCs of ARAS Pigs
Single
Apoptotic
Dead
Live
Intensity_MC_11_AnnV
Intensity_MC_2_Sytox
1e8
1e7
1e6
1e5
1e4
1e3
100
0
-100
-1e3
-1e4
1e5
B
Live Cells
Apoptotic Cells
%
Healthy Normoxia
Healthy Hypoxia
ARAS Normoxia
ARAS Hypoxia
